# A single-center experience of post-transplant lymphomas involving the central nervous system with a review of current literature

**DOI:** 10.18632/oncotarget.26522

**Published:** 2019-01-11

**Authors:** Anju John John Velvet, Shiv Bhutani, Stavros Papachristos, Reena Dwivedi, Michael Picton, Titus Augustine, Muir Morton

**Affiliations:** ^1^ Department of Renal Medicine and Transplant Nephrology, Manchester Royal Infirmary, Manchester University Hospitals NHS Foundation Trust, Manchester, UK; ^2^ Department of Renal and Pancreas Transplantation, Division of Surgery, Manchester Royal Infirmary, Manchester University Hospitals NHS Foundation Trust, Manchester, UK; ^3^ Division of Diabetes, Endocrinology and Gastroenterology, Faculty of Biology, Medicine and Health, Manchester Academic Health Science Centre, University of Manchester, Manchester, UK; ^4^ Department of Radiology, Manchester Royal Infirmary, Manchester University Hospitals NHS Foundation Trust, Manchester, UK

**Keywords:** CNS-PTLD, EBV, mycophenolate, transplantation, immunosuppression

## Abstract

**Background:**

Central Nervous System (CNS) lymphoma is a rare presentation of post-transplantation lymphoproliferative disorder (PTLD).

**Methods:**

This single center retrospective study reviewed presentations, management and outcomes of CNS lymphomas in kidney transplant patients transplanted 1968 to 2015, and reviews relevant current literature.

**Results:**

We identified 5773 adult kidney transplant recipients of who 90 had a PTLD diagnosis confirmed. CNS disease was diagnosed in 6/90 (7%). Median age at presentation was 60 years and time from transplant 4.5 years. Immunosuppression at diagnosis included mycophenolate mofetil and prednisolone without calcineurin inhibitor in 5/6 patients. Histological analysis diagnosed monomorphic disease in 5/6, and one polymorphic case with tissue positive for Epstein-barr virus (EBV) in 5/6 cases. Despite this 2/4 EBV positive cases had no detectable EBV in peripheral blood or CSF at diagnosis. Treatment strategies included reduction in immunosuppression in all, chemotherapy (n=5), radiotherapy (n=3), Cytotoxic T-Lymphocytes and Craniotomy (n=2). Patient survival was 40% at 1 year with CTL treated patients surviving beyond three years from diagnosis.

**Conclusion:**

This study supports observational data suggesting MMF treated patients without CNI may have increased risk of disease. Peripheral blood screening for EBV DNAemia does not seem helpful in early identification of those at risk.

## INTRODUCTION

Lymphomas in the central nervous system are a rare presentation of PTLD. Outcomes are often poor and diagnosis made late. CNS PTLD is frequently monomorphic, B-cell in origin [1] and positive for EBV infection in up to 70% cases [2, 3, 7]. Among CNS disorders presenting after transplantation, PTLD follows cerebrovascular disease and infection in frequency and post-transplant autopsy series have found CNS PTLD in 2-7% of brains examined [4, 5]. Retrospective analyses of primary CNS PTLD presenting in SOT recipients found the greatest incidence in kidney allograft recipients (79%), followed by liver, heart, lung and pancreas [6].

Retrospective studies have suggested an increase in the incidence of Primary CNS PTLD since the introduction of new immunosuppression regimes. Crane et al investigated the association of immunosuppression in a total of 174 patients including 29 with primary CNS PTLD. The study suggested that the use of CNI may be protective and that the increasing use of MMF may be associated with an increasing incidence of EBV positive primary CNS PTLD [7]. Anecdotal review of cases presenting to our unit in recent times suggested that patients presenting with CNS PTLD were typically on MMF and prednisolone without CNI.

Guidelines recommend the monitoring of EBV DNA in peripheral blood in the first post-transplant year in high risk EBV seronegative recipients. However, there is no specific guidance on screening for EBV DNAemia in the late post-transplant period. Case reports have suggested individuals presenting with EBV positive CNS lymphoma may have no detectable EBV viremia at time of diagnosis in peripheral blood or cerebrospinal fluid [33].

A diagnosis of PTLD is generally associated with a poor prognosis due to either graft failure from reduction of immunosuppression (RI), disease progression or toxicity from therapy [8]. Registry series have published overall response rates of 60% and survival rates of 54% at 3 years and 45% survival at 10 years with treatment related mortality causing 13% [9, 6].

### Aims

To perform a single center review of presentation, management and outcomes of CNS lymphomas in kidney transplant patients transplanted from 1968 to 2015 along with a systematic review of relevant literature.

## RESULTS

We identified CNS PTLD in 6/90 (7%) patients, of which 4 patients presented with primary CNS PTLD and 2 patients presented with systemic PTLD with CNS involvement. Patients included 3 male and 3 female with a median age of 48.5 years at transplant and 59.5 year at diagnosis 59.5 years. Median time from transplant to diagnosis was 4.5 years, with 2 presenting early within 1 year of transplant and 4 late.

Primary disease, comorbidities and family history are presented in Table 1. Two patients had some previous form of low grade cancer which included Bowen's disease and anal intraepithelial neoplasia. Two had positive family history for cancer, one patient with a family history of colorectal cancer and the other recipient's first degree relative was diagnosed with Hodgkin's lymphoma.

**Table 1:** Demographics and background history

Patient	Gender	Age (at diagnosis)	Time from transplant (Years)	Primary Disease	Comorbidities	Family History
1	M	62	1	SLE	DVT, HTN, Osteoporosis	
2	F	66	20	GN	Bowen's disease, Atrial Flutter, HTN	
3	F	57	13	HTN	TB, Bronchiectasis, MGUS	First degree relative with Hodgkin's lymphoma
4	M	69	3	GN	HTN, Hyperlipidaemia	
5	M	55	6	PKD	AIN, psoriasis, HTN, stroke, cmv colitis, hypercholesterolaemia	Colorectal cancer
6	F	35	2	Bergers nephropathy	HTN, retinopathy	

Abbreviations: SLE; systemic lupus erythematosus, HTN; hypertension, GN; glomerulonephritis, PKD; polycystic kidney disease, DVT; deep vein thrombosis, TB; tuberculosis, MGUS; monoclonal gammopathy of undetermined significance, AIN; acute interstitial nephritis, CMV; cytomegalo virus.

**Figure 1:**
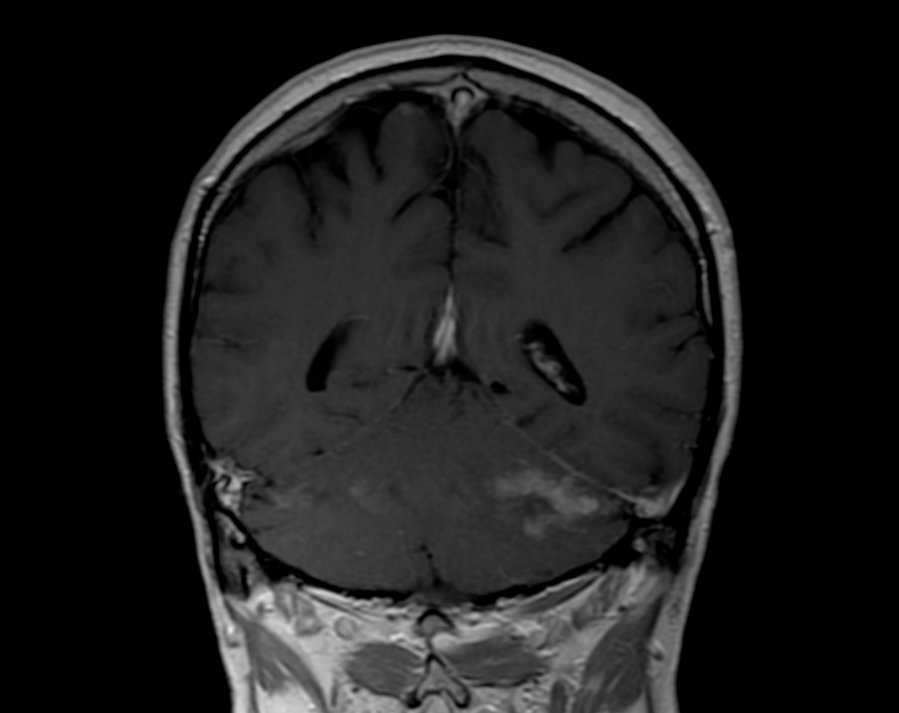
MRI brain of patient 1 before treatment showing a lesion in the cerebellum

**Figure 2:**
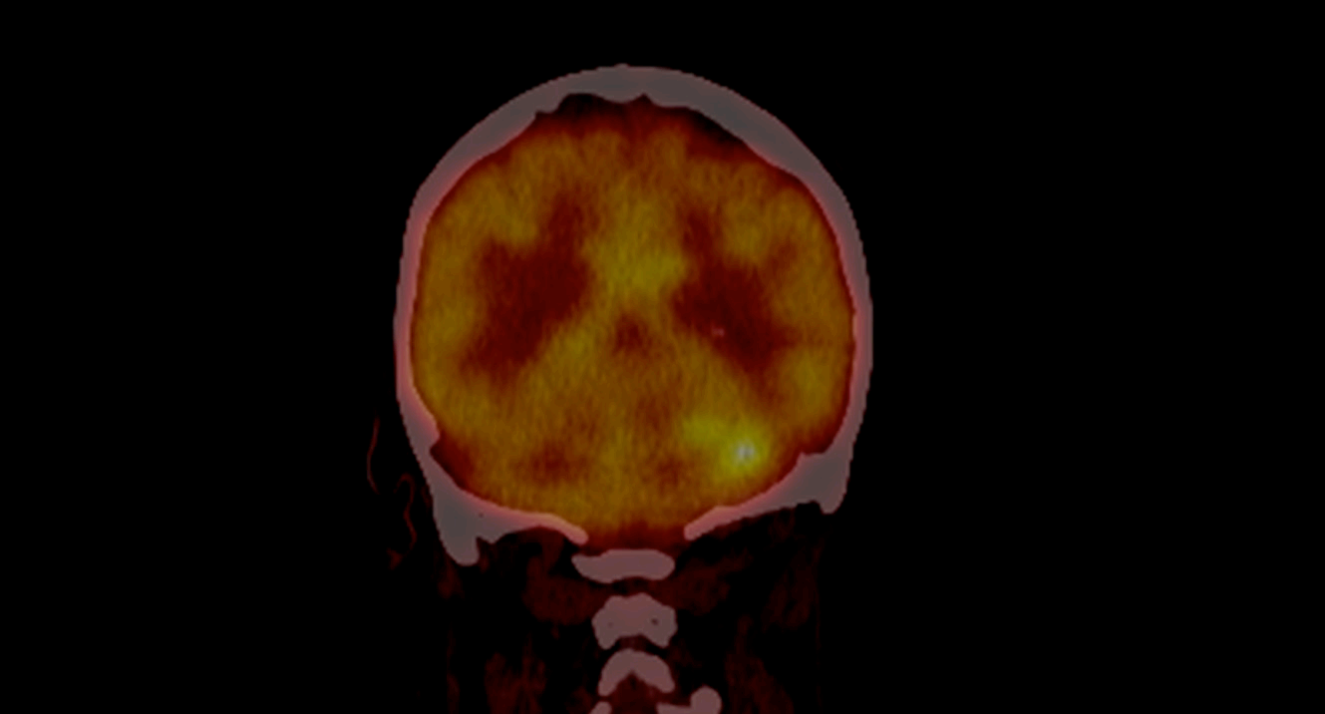
PET CT of patient 1 before treatment showing increased uptake in the cerebellum

**Figure 3:**
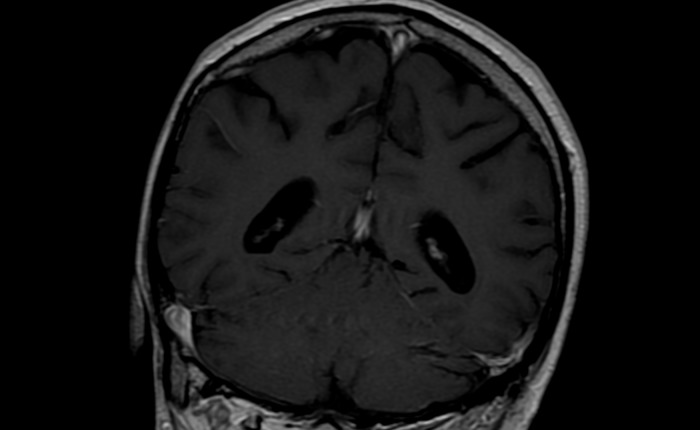
MRI brain of patient 1 shows resolution of lesion post treatment

Table 2 describes the initial presentation and prognostic indicators at the time of diagnosis. Patient 1 who was 62 years old presented initially, 3 months after transplantation, with B symptoms and lymphadenopathy. He received Basiliximab as an induction agent with no episodes of rejection requiring therapy and immunosuppression at presentation included Tacrolimus, MMF and prednisolone. The patient was EBV seronegative at transplant, and at presentation had EBV DNA detected by PCR at 13428 copies/ml (log = 4.13) consistent with primary infection. CT demonstrated nodal disease in the mediastinum, neck, right and inguinal regions. Histology showed an EBV positive diffuse large B-cell lymphoma (DLBCL), with a raised LDH of 800. PET identified metabolically active disease above and below the diaphragm. Chemotherapy was commenced with a regimen of RCHOP which led to complete remission at the sites identified on PET scanning, however 6 months after chemotherapy, PET showed a new metabolically active area in the cerebellum (Figure 2). He therefore went on to have an MRI scan with gadolinium enhancement (Figure 1). This revealed multifocal white matter lesions including the cerebellum. Neuro-radiological consensus was of PTLD. EBV DNA in both blood and CSF were not detected during the time when CNS PTLD was diagnosed. The patient received CNS radiotherapy and 4 cycles of EBV specific CTL infusion. Initial repeat MRI scan at 1 month of treatment showed improvement in infratentorial lesions (Figure 3), improvement or static change in many supratentorial lesions but development of one lesion within the right middle frontal gyrus. MRI scan 1 year later showed no further significant changes in the previously identified intracranial lesions. of the brain showed a mixed picture of ongoing CNS lesions. Clinically the patient has remained stable over 3 years with no new focal neurological symptoms, headaches or B symptoms. Further MR imaging and clinical review is awaited.

**Table 2:** The year of diagnosis and clinical, radiological and biochemistry at the time of diagnosis

Patient No	Clinical presentation	Year at Diagnosis	Radiological Findings	Albumin at diagnosis (g/L)	LDH (U/L)	Lymphocytes at diagnosis (X10^9/L)	Platelet at diagnosis (X10^9/L)
1	Cervical, Superior mediastinal, inguinal lymphadenopathy, fever, collapse and weight loss.	2015	Multi-focal white matter lesions in the frontal and cerebellar lobes.	38	734	0.44	314
2	Right maxillary swelling.	2011	Focal abnormality in the right temporal region.	36	739	7.3	223
3	Periods of depressed mood, tearfulness, memory impairment and prosopagnosia. Confusion, memory loss, headache and tingling in fingers and shoulders.	2014	12*9 mm ring enhancing lesion within left frontal lobe, white matter edema in frontal, parietal and temporal lobes.	28		0.52	147
4	Truncal ataxia, dysphasia, incontinence and collapse.	2008	Multiple intrinsic enhancing brain lesions in the left cerebellum, right occipital, frontal and parietal lobes.	39	421	1.75	252
5	Spasms, twitches, abnormal gait, global weakness, memory impairment, expressive dysphasia, confusion, anorexia, weight loss and drenching night sweats.	2007	Multiple cerebral lesions in left fronto-parietal lesion, high parietal region and right parafalcine area.	35		0.52	210
6	Vertigo, double vision, right side hearing loss and cerebellar symptoms.	2004	4 lesions with involvement of the right cerebellum, the left posterior frontal area and the right posterior parietal region.	47		2.12	284

The second patient was 66 years old at the time of diagnosis, was initially on Tacrolimus monotherapy as maintenance immunosuppression which was then substituted with MMF and prednisolone due to deteriorating graft function and had a biopsy showing CNI induced toxicity in the graft. Tacrolimus was withdrawn approximately 11 months prior to PTLD presentation. The patient then presented with a non-healing ulcer in the oral cavity with biopsy showing a high grade oral lymphoma and a subsequent focal abnormality in the right temporal region, about 20 years after transplantation. EBV DNA was detected in whole blood by PCR at 1085 copies/ml. Brain biopsy was not performed and CNS PTLD was diagnosed on the basis of the radiological findings with the oral cavity biopsy.

Patient 6 presented 6 weeks post-transplant with decline in kidney function and a kidney biopsy showing micro thrombi within arterioles attributed to Ciclosporin. She was treated with high dose IV methyl prednisolone, cyclosporine was stopped and MMF was introduced. She subsequently remained on MMF and prednisolone for approximately 16 months when she presented with CNS symptoms and MRI brain showing multiple lesions and biopsy consistent with diffuse large B-cell lymphoma.

Two out of these six patients had characteristic ring enhancing lesions on cross-sectional imaging. Brain biopsy was performed in 4 patients of which 3 showed DLBCL (2 EBV positive, 1 unknown), while one patient was diagnosed with EBV positive polymorphic PTLD. Patient 1 and 2 had no brain biopsy and the basis for their CNS lymphoma diagnoses described above but had EBV tissue positive PTLD. All cases in this series were identified after 2004. As per the WHO classification of PTLD, 5 patients in our cohort presented with diffuse large B-cell Lymphoma, out of which one patient had tissue diagnosis from his inguinal lymph node and no brain biopsy. Case 4 was given an initial diagnosis of Diffuse-large B cell lymphoma but subsequent review by the Hematological malignancy diagnostic service (HMDS) concluded an EBV positive polymorphic PTLD. Case 3 also had review by the HMDS and case 6 a second opinion from a specialist haemato-oncology pathologist. Cases 1 and 2 did not have brain biopsy performed. Case 1 had PTLD diagnosed on the basis of his inguinal lymph node biopsy and subsequent radiological and clinical findings in relation to his brain. Case 2 had cerebral PTLD diagnosed on basis of biopsy of maxilla showing PTLD and radiological findings consistent. PTLD diagnoses in these last 2 cases were based on HMDS review. Despite EBV positive tissue (n=5 tested), EBV DNA was only mildly positive (1000 copies/ml) in blood (PCR) at the time of diagnosis in 2 out of 5 and negative in 3 patients. EBV was not detected in the CSF of any patients who had lumbar puncture performed (n=3) although 2 had EBV viremia at the time and all had EBV positive PTLD.

### Treatment

All cases presented were diagnosed after 2004 and all cases except 1 fit the classification of diffuse large B cell lymphoma in the current WHO classification. The authors acknowledge that treatment strategies continue to develop and can only report the strategies used for the patients who are presented. Treatment of lymphoma included reduction in immunosuppression in all patients, chemotherapy (n=5), radiotherapy (n=3), and Craniotomy (n=2). N=2 received EBV specific CTLs with good resolution of lesions in the MRI scans post treatment. Table 3 shows the treatment received, outcome and the cause of death.

**Table 3:** Treatment received

	Treatment	Outcome - Patient Survival	Cause of Death
1.	RIS, Chemotherapy, Radiotherapy, 4 cycles of CTLs	Mixed response, has a new CNS lesion with resolution of previous lesions, probable ongoing disease and alive 3 years post diagnosis.	Alive
2.	RIS, systemic and intrathecal chemotherapy	Died. 25 days	Chest Sepsis
3.	RIS,	Died. 2 months	Advanced Lymphoma
4.	RIS, craniotomy, radiotherapy, rituximab.	Died 5 months Graft failure requiring hemodialysis,	Acute Left ventricular Failure
5.	RIS, radiotherapy, chemotherapy.	Died 3 years	unknown
6.	RIS, 8 cycles of CTLs	Died 9 years	Pulmonary Embolism

Abbreviations: RIS; reduction in immunosuppression, CNS; central nervous system, CTLs; cytotoxic T lymphocytes.

### Outcomes

Patient survival at 6 months from diagnosis was 66.6%, 1 year 40% and 5 years 20% with median of 5 months duration from diagnosis to death in 5/6 patients. Death censored graft loss occurred in 1 patient who returned to dialysis. The two patients who presented within 1 year of transplantation showed a better response to treatment and had a better survival. The above two patients also received CTLs showing a better response to treatment with one of them still alive at 3 years post diagnosis and the other patient passed away 9 years after the diagnosis, with cause of death being recorded as due to pulmonary embolism.

## DISCUSSION

Malignancy is the result of a multifactorial process with complex interlinking, predisposing and modulating mechanisms. There is an increased 2-3 fold cancer risk after solid organ transplantation when compared to the general population [10]. The innate protective mechanisms and barriers of a competent immune system through the action of CTLs, macrophages and natural killer cells are silenced by immunosuppression required to prevent rejection of the transplanted organ. This disruption of the immune system promotes mitotic transformation of cells and allows them to escape immune recognition. [11]

PTLD has a bimodal presentation with a high risk period identified during the first year in seronegative recipients but the period of greatest incidence is in the late post-transplant period for CNS PTLD [12]. Report of a large nationwide French registry showed early onset(<12 months) in 14% this included systemic and CNS PTLD [9]. CNS PTLD in the large case series published to date suggest the period of greatest incidence is at approximately 5 years post-transplant where among 84 PCNS PTLD median time from transplant to PTLD diagnosis was 54 months [6].

A pathological diagnosis is based on the WHO classification which has four major categories-early lesions, polymorphic PTLD, monomorphic PTLD which includes the DLBCL and classical Hodgkin lymphoma type PTLD [13]. However, in clinical practice it usually broadly varies between early lesions, polymorphic and monomorphic PTLD [13]. The use of increased immunosuppression at the first year of transplant or immediately after organ rejection increases the risk of developing “early PTLD” [14]. With a preservation of the tissue architecture there are two “Early” histological patterns that are identified-plasmacytic hyperplasia and Infectious Mononucleosis like lesion, showing EBER positivity on immuno-phenotype and oligo clonal or polyclonal EBV genome on molecular analysis [13]. Our study identified two patients who presented “early” with CNS lymphoma within the first year of transplantation, with the other patients presenting “late” from 3 years to about 20.35 years after transplantation. Early PTLD is usually EBV positive, involves the allograft in 57% and may respond well to RI. Late PTLD is usually monomorphic, more often EBV negative, less frequently involves the allograft and may respond less to RI.

As per the WHO classification of PTLD, 5 patients in our cohort were given a pathological diagnosis of monomorphic diffuse large B-cell Lymphoma, out of which one patient had tissue diagnosis from his inguinal lymph node and no brain biopsy. Another patient had a diagnosis of polymorphic PTLD which was confirmed by HMDS review.

DLBCL is one of the most common lymphoproliferative disorders that occurs in an immunocompromised setting and one third of these cases are related to EBV infection. DLBCL is usually rapidly growing and its symptoms are most likely related to secondary mass effect [15].

Clinical presentations corresponded to the area of the brain involved along with general symptoms like fever, weight loss, night sweats, mood disturbances, tearfulness, memory disturbances and confusion. MRI and a positive histopathological diagnosis of PTLD is the gold standard in the diagnosis of PTLD [2] with diffusion-weighted imaging MRI playing an important role for evaluating treatment response [16].

The presence of EBV infected tumor cells in a high proportion of the PTLD population highlights the important role that EBV has to play in the development of post-transplant lymphomas [17]. In our study, 4 patients were found to have positive EBV LMP and EBER-ISH in the diseased tissue obtained from brain biopsy; one patient had no examination of the tissue for EBV and a further patient with EBV positive PTLD in a inguinal lymph node biopsy. The incompetence of the immune system in controlling latent EBV infection is the original cause of EBV-positive PTLD [18]. Transplant patients receiving high amounts of immunosuppression causing severe T cell immunosuppression allows EBV infected B cells to be transformed and immortalized, and multiply causing malignant lymphoproliferation [19]. A prospective study in heart and lung transplant patients looking at the correlation of EBV levels and clinical events shows that during the first 9 months post-transplant is the period when no CTL activity was detected in any patients due to high immunosuppressive therapy to prevent any acute rejection and this is when the detection of peripheral blood EBV levels are very high at about 80% and the incidence of early PTLD is very high [20]. EBV was detected in 85% of throat washings (TW) of patients in 3-6 months which then decreased to 71% in 6-12 months and 65% in 12-24 months [20]. A paper by Morton et al showed that of well adult kidney transplant patients screened for EBV viremia in the late post-transplant period (beyond 1 year) had detectable viremia including persistent viremia but no PTLD [21]. Also 2/6(33%) PTLD cases occurred in seronegative recipients and a patient with tissue negative for EBV had persistent EBV DNAemia during the sampling period, however during the time of PTLD diagnosis it was found to be negative in blood [21]. In addition the study demonstrates an association of detectable EBV levels and the use of MMF, this is suggested to be due to the reduction of EBV carrying B cell population [21]. The study advises that high risk seronegative recipients should receive equal attention on screening for symptoms and clinical examination along with monitoring EBV DNA levels as 50% of late PTLDs have a EBV negative diagnosis in adult renal transplant patients [21]. CNS PTLD is associated with >90% EBV positivity by EBER *in situ* hybridization which is more than the association of EBV with systemic PTLD [22]. However, only 27% were positive for detectable levels of EBV DNA in peripheral blood at the time of diagnosis. This obvious discrepancy between undetectable EBV DNA levels in blood and EBER positivity in tissue is mostly put down to the disease being more localized to the CNS with minimal systemic involvement causing low peripheral EBV DNA levels [22]. An extensive analysis evaluating the differences in the association of EBV with CNS and systemic PTLD by studying the expression of latent and lytic EBV transcripts and viral and cellular micro RNA in 9 EBV positive CNS PTLD and 16 EBV positive systemic PTLD identified 28 different microRNAs between systemic and CNS PTLD [23]. The activation of lytic replication or reactivation of the latent phase is what causes the EBV infected B cells to activate their growth program and differentiate and then the infected B cells are released into the blood stream, making it possible to detect levels of virus in the blood stream. An analysis of latent and lytic intra-tumoral EBV infection in a 35 patient series showed a low correlation between PCR in blood and replication in the involved organs [17].

Patients in our study who had EBV detected in the biopsy samples still did not show positivity in the CSF samples or blood samples at the time of PTLD diagnosis. This indicates that only monitoring EBV PCR levels in the blood might not be helpful in diagnosing suspected CNS disease. Table 4 describes the histology, EBV status pre transplant and at the time of diagnosis. A study conducted by the Helsinki University Central Hospital, looked into the EBV DNA in CSF and EBV positivity was found to be the lowest in CNS lymphoproliferative disorder along with meningitis when compared to other CNS disorders like encephalitis and brain abscess which showed increased EBV DNA positivity in CSF [24]. The specificity and positive predictive value of EBV PCR in detecting CNS-PTLD is low and monitoring viral load alone would not help in the early diagnosis of CNS-PTLD [2]. EBV naïve individuals receiving an EBV positive organ and younger groups are at particular risk for PTLD [10].

**Table 4:** Histology and EBV status at the time of diagnosis

	Histology	EBV status Pre-Transplant (Recipient)	EBV status in Tissue	EBV status in Blood	EBV status in CSF
1.	Diffuse large B cell Lymphoma	Negative	No CNS biopsy (Positive Inguinal LN biopsy)	Positive	Negative
2.	Diffuse Large B-Cell Lymphoma	Positive	No CNS biopsy (Positive maxillary biopsy)	Low level Positive	Negative
3.	Diffuse Large B-Cell Lymphoma	Unknown	Positive	Negative	Negative
4.	Polymorphic	Unknown	Positive	Low level Positive	Not done
5.	Diffuse Large B-Cell Lymphoma	Positive	Unknown	Negative	Not done
6.	Diffuse Large B-Cell Lymphoma	Unknown	Positive	Negative	Not done

Abbreviations: CNS; central nervous system, LN; lymph node.

French registry data analyzing 500 adult kidney transplant patients with PTLD between 1998-2007 developed prognostic factors for PTLD using Kaplan–Meier and Cox analyses. CNS localization was one of the independent prognostic indicators of poor survival, with an adjusted hazard ratio of 2.65 on multivariable analysis along with age >55 years at diagnosis, late onset PTLD, high LDH and creatinine levels, widespread PTLD, T cell lymphoma and monomorphic histology associated with a poor prognosis [9].

In our study, it is interesting that at the time of the diagnosis 5/6 patients were on only MMF as a mono-therapeutic agent of immunosuppression. Table 5 describes the immunosuppression regimes our patients received at induction and as maintenance. These patients previously were exposed to tacrolimus or cyclosporine but were changed or maintained on MMF due to reasons such as chronically disordered graft function, CNI toxicity or a rise in creatinine. MMF is a selective inhibitor of inosine monophosphate dehydrogenase, which is the rate limiting enzyme important for leukocyte production. Therefore, it inhibits the proliferation of T and B lymphocytes. EBV positive DLBCL involving the CNS has been reported in the use of MMF for autoimmune disorders like systemic lupus erythematosus [11]. There are few case reports which describe the development of CNS PTLD in four patients where MMF was used for other conditions other than transplant [25]. In a retrospective study that looked at patients developing late CNS PTLD, 6/10 patients had a change in their immunosuppression regimen from azathioprine to MMF just before PTLD presentation [26]. In a retrospective review of all PTLDs diagnosed at the John Hopkins Hospital, it was found that patients with CNS PTLD were more likely to have been taking MMF (15/16) when compared to non-CNS PTLD (37/102) in the year prior to or at the time of diagnosis [7]. In addition, a larger dataset from the UNOS-OPTN showed that 66.7% of the PCNS PTLD cases were in patients with MMF with no CNIs and 1.7% in those with a CNI and no MMF. This increases the odds for patients on MMF without CNIs to develop PCNS by about 118 folds compared to non-CNS PTLD, than patients on CNIs alone (95%CI, 8.7–1597; p < 0.001) [7]. Analysis of the UNOS-OPTN dataset using Firth's penalized regression method showed that the odds of PCNS PTLD were significantly increased when taking MMF as compared to not taking MMF (odds 2.9; 95% CI, 1.7–5.1; p < 0.002). CNIs significantly reduced the odds of PCNS disease compared to not taking CNIs (odds 0.34; 95% CI, 0.20–0.58; p < 0.001) [7]. These findings suggest that CNS may have an inherent susceptibility to lymphoproliferative disease in the context of immunosuppression, and the specific immunosuppressive regimen affects the likelihood of PCNS disease. CNIs, when given alone or in combination with MMF, appear to protect against the development of PCNS PTLD. Thus, our observed rise in the incidence of PCNS PTLD is likely not only due to immunosuppression with MMF but also due to a decline in the usage of the protective CNIs [27, 26]. This shows the importance of immunosuppression regimes having a role to play in the development of CNS PTLD.

**Table 5:** Immunosuppression received and its duration

	Induction	Primary immunosuppression	Immunosuppression at Diagnosis	MMF dose (mg)	Period of CNI exposure	Duration on MMF
1.	Basiliximab	Tacrolimus, MMF and prednisolone	MMF, Tacrolimus and Prednisolone	720	3 months	3 months
2.	Nil	Tacrolimus monotherapy	MMF and prednisolone	1500	9 years	1 year, 11 months
3.	Nil	Ciclosporin	MMF and Prednisolone	1000	6 years	6 years
4.	Basiliximab	Tacrolimus	MMF and Prednisolone	250	unknown	unknown
5.	Nil	Ciclosporin	MMF and Prednisolone	1000	unknown	unknown
6.	Nil	Ciclosporin and prednisolone	MMF and prednisolone	1500	4 months	16 months

Abbreviations: MMF; mycophenolate mofetil.

The challenge in treating PTLD of the CNS is that most of the chemotherapeutic agents do not cross the blood brain barrier. High dose methotrexate has been found to be useful in treating CNS PTLD [28]. Isolated CNS PTLD could be treated with radiation therapy alone, which has been effective in 85% of cases but with a median survival of just 26-36 months [27] and a five year survival of <5%^2^. Adult patients with recurrent disease after chemotherapy have used whole brain radiation therapy as a salvage treatment [2].

In a prospective study of 43 patients where rituximab was used to treat B-cell systemic PTLD with an exclusion of CNS involvement, showed an overall response rate of 40% with a one year survival rate of 67% [29]. An international multi-center open-label phase 2 trial which included 70 patients with systemic PTLD supports the sequential treatment using rituximab followed by CHOP as a first-line to all patients who do not respond to a reduction in immunosuppression [30]. Rituximab being a 145-kDa molecule cannot readily cross the blood brain barrier, with a CSF concentration of only 0.1% of the systemic level [2] and might not be effective in the treatment of CNS-PTLD. In the face of this disadvantage Bonney et al successfully treated two children with isolated CNS PTLD with intrathecal rituximab [27, 2]. In a study that looked at the efficacy of intrathecal rituximab in 8 children with CNS-EBV-PTLD showed that 7/8 patients responded well with just 2 patients showing persistent changes on their MRI brain scan [31]. However, this needs further large trials to confirm the efficacy of using intrathecal rituximab in the treatment of CNS PTLD.

A reduction in immunosuppression in established systemic PTLD aims at restoring an immune response to EBV and this could induce a tumor regression in 50 % cases without any additional therapies, especially in polymorphic forms of PTLD [32]. However in case of a late onset monomorphic PTLD which is usually EBV negative, a RI seems to be ineffective [33]. When it comes specifically to CNS PTLD patients receive aggressive treatment modalities therefore management with just a reduction of immunosuppression has not been evaluated extensively. In a case series of 34 patients, 2 patients received only a reduction in immunosuppression. One of whom remained alive at 89 months with a complete response in a follow up MRI and the other to 7.4 months [32].

All of our patients were treated with a RI. RI comes with an increased risk of rejection, HLA sensitization and the need for transplant nephrectomy [10]. Indicators of poor response to RI are bulky disease, LDH >2.5 times the upper limit of normal, organ dysfunction and the involvement of multiple sites. There is always a risk of graft rejection in up to 39% of solid organ transplants with the RI [34]. 1/6 of our patients developed biopsy proven graft rejection requiring dialysis. A study has shown that holding immunosuppression while the patient is on chemotherapy and restarting RI was found to be safe in terms of graft outcomes [15].

2/6 patients who presented with early CNS PTLD in our study received EBV specific CTLs which showed a good resolution of the lesions in the MRI scan done post treatment. EBV specific CTL therapy has been found to be useful in EBV positive PTLD even when they are resistant to rituximab. However, the facilities and experience in using it is not widely available [15, 22] and there is always a risk of graft versus host disease with allogenic T cells [15].

## MATERIALS AND METHODS

Ninety adult patients with a diagnosis of PTLD were identified in a single center. Retrospective case based review was performed of identified cases. Diagnosis was established from the clinical presentation, radiological and pathological findings. We analyzed patient demographics, primary disease, immunosuppression at the time of transplantation and diagnosis, clinical presentation, time of clinical presentation after kidney transplantation, timing of diagnosis after kidney transplantation, treatment received and both graft and patient survival. In addition detection of EBV in histological specimens, peripheral blood and CSF was analyzed in patients with disease. The histological specimens for 3/6 patients had a subsequent review by the Hematological malignancy diagnostic services (HMDS) in Leeds. We also looked at the duration of CNI exposure and MMF monotherapy where known, LDH, Albumin, Lymphocyte, platelet levels pre diagnosis to identify any prognostic factors. Informed consent was obtained from living patient and patient identifiable information not included in the manuscript.

## CONCLUSIONS

In our series CNS PTLD is uncommon involving only 7% cases, and presents late (median 4.5 years). Histology is typically DLBCL and EBV positive yet blood and CSF samples may have undetectable or low level EBV viral loads at diagnosis. Further studies on targeting EBV associated pathways in the pathogenesis needs to be encouraged which would introduce different treatment options in the future. We observed a high prevalence of patients on MMF at diagnosis with prior CNI withdrawn. Further research is needed to look into the mechanism of action of immunosuppressive regimes that might make patients susceptible to developing a CNS-PTLD.
